# Association between joint hypermobility, scoliosis, and cranial base anomalies in paediatric Osteogenesis imperfecta patients: a retrospective cross-sectional study

**DOI:** 10.1186/1471-2474-15-428

**Published:** 2014-12-13

**Authors:** Heidi Arponen, Outi Mäkitie, Janna Waltimo-Sirén

**Affiliations:** Department of Orthodontics, Institute of Dentistry, University of Helsinki, PO Box 41, FI-00014 Helsinki, Finland; Children’s Hospital, Helsinki University Central Hospital and University of Helsinki, Helsinki, Finland; Folkhälsan Research Center, Helsinki, Finland; Center for Molecular Medicine, Karolinska Institutet, and Department of Clinical Genetics, Karolinska University Hospital, Stockholm, Sweden

**Keywords:** Osteogenesis imperfecta, Joint hypermobility, Scoliosis, Craniovertebral pathology

## Abstract

**Background:**

Joint hypermobility is a common clinical characteristic of patients with Osteogenesis imperfecta (OI), a disorder with serious comorbidities of scoliosis and cranial base anomalies. This study aimed at evaluating how prevalent joint hypermobility is in paediatric OI patients, and to find out whether it serves as a potential predictive marker of the different spinal complications; scoliosis and craniovertebral anomalies (basilar impression and basilar invagination).

**Methods:**

In this cross-sectional one-center study we analysed retrospectively clinical patient records and radiographs of 47 OI patients, aged 1–19 years, some of whom were treated with bisphosphonates. Presence of joint hypermobility, scoliosis, and craniovertebral anomalies was recorded and possible connections between the phenomena were explored with correlation analysis.

**Results:**

Joint hypermobility was found in 70% of the patients. Scoliosis and cranial base anomalies had developed in 26%. The presence of spinal complications was independent of the bisphosphonate treatment status and joint hypermobility.

**Conclusions:**

Scoliosis and craniovertebral anomalies are strongly associated in paediatric OI patients. Joint hypermobility that is much more common appears, however, to be a poor predictor.

**Electronic supplementary material:**

The online version of this article (doi:10.1186/1471-2474-15-428) contains supplementary material, which is available to authorized users.

## Background

Osteogenesis imperfecta (OI) is a heterogeneous group of rare inherited connective tissue disorders of bone fragility due to abnormal collagen composition. In addition to bone, other tissues rich in collagen, including ligaments, skin, and cornea, may be affected. OI is characterized by osteopenia and fractures, short stature, and excessive ligament laxity[1]. Classification of OI is based on the causative gene mutation and/or clinical and radiological phenotype[2–4].

Joint hypermobility is defined as a range of movement exceeding the normal level for age, sex, and ethnic background[5]. The maximum range of movement of a joint is determined by the morphology of the bones, tightness of the capsule, and ligaments attached. The primary cause of hypermobility is ligament laxity[5]. Females tend to be more lax than males, and the degree of laxity decreases with age[6]. In European paediatric population general ligament laxity is considered to be present in 5–35% of children[7, 8]. Hypermobility is commonly diagnosed with Beighton score that evaluates the ability to perform a selection of manoeuvers involving five different joints[6, 9]. Various degree of joint hypermobility is seen for instance in different types of Ehlers-Danlos syndrome which, alongside OI and Marfan syndrome, form a heterogeneous group of heritable connective tissue disorders. In addition to each possessing characteristic symptoms, considerable overlap exists in their clinical phenotype, such as skin hyperextensibility and joint hypermobility. The latter is a congenital feature that has been linked with instability of the skeleton and with scoliosis[5].

Scoliosis is a common problem in OI, and affects about half of the patient population[10, 11]. One of the most serious complications of OI is craniocervical pathology in the form of basilar impression or basilar invagination. The condition may be asymptomatic or lead to compression of the brain stem and/or related neurovascular structures, resulting in a variety of subsequent neurological complications and symptoms[12]. Platybasia (flattening of the anterior cranial base) has been associated with craniocervical junction pathology[13–15]. Early recognition of patients at risk of developing craniocervical pathology and/or progressive scoliosis is essential for the treatment planning. Our aim was to evaluate how common joint hypermobility is in paediatric patients with OI and to find out whether it serves as a potential predictive marker of spinal pathology in the form of scoliosis or craniovertebral anomaly.

## Methods

This retrospective cross-sectional one-center study was carried out at the Children's Hospital, Helsinki University Central Hospital, Finland. The study was approved and consent for the use of patient records was given by the Research Ethics Committee, Children’s Hospital, Helsinki University Hospital. The study group consisted of patients with OI, who are or have previously been under follow-up at the Metabolic Bone Clinic, Children’s Hospital during the years 1996–2013. The inclusion criteria were 1) diagnosis of OI, 2) availability of radiographic data on the cranium, and 3) age between one and 19 years at the time of imaging.

Altogether 47 patients fulfilled the inclusion criteria. Their patient files were reviewed to obtain data on the OI type, presence of joint hypermobility, spinal deformity, bisphosphonate treatment history, and neurological status. Classification of the OI type was based on clinical and radiological features as well as available genetic data, and grouped as mild, moderate, or severe. When radiological longitudinal follow-up data were available (25 patients), the most recent radiograph and medical record notes at the time point were included in the cross-sectional data. The height measurements of the patients were converted to age- and sex-specific Z-scores according to Finnish references[16].

The decision of bisphosphonate treatment was made individually based on the severity of the disorder and symptoms. According to local guidelines, the patients were eligible for treatment if they had (1) a history of frequent fractures, (2) vertebral compression fractures, (3) chronic disabling bone pain, and/or (4) their vulnerability to fractures was estimated high. Both intravenously and orally administered bisphosponates have been used for treatment of OI in Finland since the year 2001.

Diagnosis of scoliosis was made on the basis of clinical and radiological findings by a paediatrician and/or paediatric orthopaedic surgeon. In clinical examination, the angle of trunk inclinations was recorded in forward bending position with a scoliometer at the upper thoracic (T3-T4) and at the thoraco-lumbar (T12-L1 or L2-L3) levels. Radiographs of the spine were obtained when severe scoliosis or kyphosis was suspected based on clinical examination. Scoliosis was recorded when the spinal inclination exceeded 6 degrees as measured with a scoliometer or 10 degrees in radiographic evaluation of the coronal plane[17]. Diagnosis of joint hypermobility was made based on clinical findings of the large joints by a paediatrician and/or paediatric orthopaedic surgeon. Patients with Beighton score of four out of nine or higher were diagnosed as hypermobile. Diagnosis of cranial anomaly was done from mid-sagittal skull radiographs based on three previously documented reference line measures: 1) McRae measure to diagnose the presence of basilar invagination, 2) D-M angle to diagnose basilar impression, and 3) anterior cranial base angle to diagnose platybasia. An anomaly was present when McRae measure was at or above 0, or either of the angular measure values exceeded 2.5 SD from that of age-specific norms, thus indicating an abnormally high position of the odontoid process or an abnormally flat cranial base[18].

For statistical analysis, presence of joint hypermobility, scoliosis, and cranial anomalies were classified as dichotomous variables (yes/no). Associations between the variables were computed with phi correlation coefficient. Association between joint hypermobility and height Z-score was computed with point-biserial correlation coefficient (SPSS software version 19; SPSS Inc., Chicago, IL, USA).

## Results

The majority of the patients (64%) represented mild OI (type I), 21% had moderate OI (types IV and VI), and 15% had severe OI (type III). Their mean height Z-scores were in line with the general severity of the disorder as documented in previous studies[19]. Joint hypermobility was present in 70% of patients, and scoliosis in 26% of patients. Of the patients, 26% (12 patients) had at least one form of cranial anomaly (basilar impression, basilar invagination, or platybasia). Nine patients exhibited platybasia, eight patients exhibited basilar impression, and seven patients basilar invagination.

**Figure 1:**
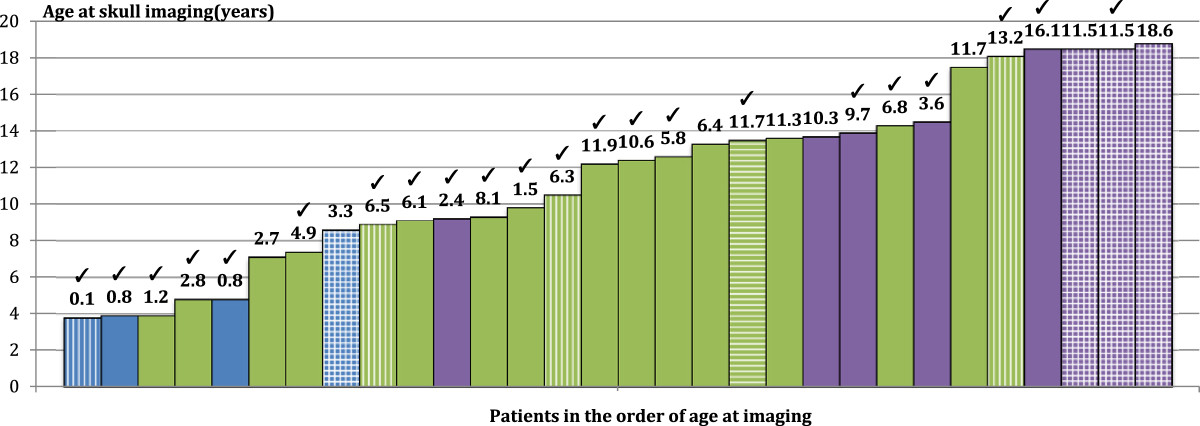
**Data on the bisphosphonate-treated patients (n = 30); green color indicates mild OI type, purple color moderate type, and blue color severe OI type.**

The prevalence of joint hypermobility was independent of the severity of OI, whereas scoliosis and cranial anomalies were more common findings in the moderate and severe OI types than in the mild OI type (Table 1). The results of the phi coefficient analysis on the whole patient material indicated no association between joint hypermobility and scoliosis (Phi = -0.152, p = 0.30, n = 47) or between joint hypermobility and cranial anomalies (Phi = -0.221, p = 0.13). No association was found between joint hypermobility and height Z-score (r_pb_ = 0.113, p = 0.45, n = 47). A significant positive correlation was, however, detected between scoliosis and presence of some form of cranial anomaly (Phi = 0.511, p = 0.00).Of the 47 patients, 30 (64%) had been treated with bisphosphonates. Most of the patients were treated with intravenous bisphosphonates (pamidronate and zoledronic acid) and only 2 patients received an oral preparation (risedronate). The average treatment time was 4.3 years (range 0.2 – 10.8 years) prior to cranial imaging. Characteristics of the patients treated with bisphosphonates are depicted in Figure 1. In this subgroup of patients, the results of the phi coefficient analysis indicated similarly no association between joint hypermobility and scoliosis (Phi = -0.04, p = 0.81, n = 30), or joint hypermobility and cranial anomalies (Phi = -0.005, p = 0.98). A significant positive association was again detected between scoliosis and the presence of a cranial anomaly (Phi = 0.92, p = 0.00).

**Table 1:** **Summary of the OI patient cohort, medication data, and findings**

	Mild (type I)	Moderate (types IV, VI)	Severe (type III)	Total
Number of patients	30	10	7	47
Age range at skull imaging (years)	1.3-18.1	9.2-18.8	3.8-8.6	1.3-18.8
Mean height Z-score	-0.8	-4.0	-5.4	-2.2
Number of patients treated with bisphosphonates	18	8	4	30
Mean duration and range of bisphosphonate treatment prior to imaging (years)	3.6 (0.1-8.3)	3.2 (0.2-10.9)	3.2(2.2-4)	0.7 (0.1-10.9)
Number of patients with hypermobile joints	22 (73%)	6 (60%)	5 (71%)	33 (70%)
Number of patients with scoliosis	3 (10%)	5 (50%)	4 (57%)	12 (26%)
Number of patients with cranial anomaly	2 (6.7%)	5 (50%)	5 (71%)	12 (26%)

Of the 25 patients with longitudinal follow-up data on skull base morphology, 19 patients (76%) had been treated with bisphosphonates. The six patients that had not received bisphosphonates were treated in the Children’s Hospital at a time when bisphosphonates were not generally used in the treatment of OI. Of the patients treated with bisphosphonates, four had developed platybasia and scoliosis by the ages of 7, 8, 13.5, and 18 years, respectively, and one had developed only scoliosis by the age of 9 years during the follow-up. The youngest patient diagnosed with scoliosis was 3.8 years at the time of the diagnosis. She had severe OI and had been treated with bisphosphonates since one month of age and was able to sit supported in a wheelchair. Four of these six patients that developed scoliosis despite bisphosphonate treatment also had hypermobile joints. Of the six patients that had not received bisphosphonates three developed scoliosis at the ages of 4, 9, and 9 years. One of them also had hypermobile joints. In total, of the 25 patients with longitudinal follow-up, 16 were diagnosed with joint hypermobility (64%) and 14 (56%) developed a spinal complication. Hypermobility and spinal complications co-emerged in six patients.

## Discussion

Patients with OI commonly present with varying degree of ligament laxity. We found joint hypermobility to be such a frequent finding that it could not serve as a predictive marker for the more rare spinal complications, and it was equally prevalent in mild and severe types of OI. The prevalence of joint hypermobility in patients with OI, not treated with bisphosphonates, has previously been reported to be 34%[10]. In our patient material, with a similar age range, joint hypermobility was more common with a prevalence of 70%. Our findings are consistent with those of Brizola and co-workers, who found joint hypermobility in 58 – 100% of paediatric patients, most of whom had been treated with intravenous bisphosphonates[20]. The group used a Beighton score of five as the cut-off value. Several studies have indicated that bisphosphonate treatment reduces the number of fractures in children with OI[21, 22]. Thus the reported smaller prevalence of joint hypermobility in the untreated patients might be attributed to relative or actual joint stiffness caused by numerous bone fractures and deformities. This hypothesis is supported by the reported generalized reduction in joint range of motion in untreated patients with moderate and severe OI[23, 24].

The prevalence of spinal deformity in children with OI has been documented to range between 39 and 100%[10, 25, 26]. The aetiology of the spinal curvatures remains disputable. Vertebral fractures and deformity, as well as muscular weakness and ligament laxity, are all believed to contribute to the development of spinal deformity[11], althoughscoliosis and basilar impression have been documented to be less frequent in children with OI and generalized joint hypermobility than in those with normal joints[10]. Based on this, Engelbert and co-workers suggest that microfractures of the vertebrae play an important role in the development of spinal complications[10]. Contrary to this conclusion, other studies have proposed laxity of spinal ligaments to result in vertebral instability and thus underlie progressive spinal curvature[27, 28]. Moreover, vertebral body shape has been outlined as a predictor of spinal deformity in patients with OI[26].

Scoliosis is more common in the more severe forms of OI and a rare finding in patients under the age of five, and[27, 29]. After the age of six, a rapid progression of scoliosis is observed in patients not treated with bisphosphonates[27]. Our findings are in agreement with these in that only two children both suffering from severe OI developed scoliosis under the age of six years.

Cranial base anomalies have been found to be present in more than one third of patients with OI regardless of bisphosphonate treatment status[18, 30]. In children with OI, craniocervical pathology is an acquired anomaly that has been detected from the age of two years, corresponding with the delay in age at which a child with a severe form of OI attains an upright position[19, 23, 31]. Children with mild form of OI, on the other hand, reach motor milestones at a rate close to that of healthy peers[32]. The pathogenesis of cranial base anomalies and the factors predisposing to their development are unknown. Basilar impression has been suggested to be more commonly associated with kyphosis than with scoliosis in paediatric OI patients not treated with bisphosphonates[10]. In this study, seven out of the eight patients with basilar impression also displayed basilar invagination and/or platybasia.

Very little published data are available on craniocervical anomalies in other syndromes of joint hypermobility that would shed light on the pathogenesis of craniocervical anomalies. Prevalence of basilar impression in patients with Marfan syndrome has been stated to be 36%, and prevalence of focal kyphosis 16%[33]. Nevertheless, in only 11% of the patients with Marfan syndromethe tip of the odontoid extended a minimum of 7 mm above Chamberlain’s line, which is the upper limit found in normal population and the criterion of basilar impression is possibly fulfilled due to a morphological bone anomaly of enlarged odontoid. Patients with Marfan syndrome have been observed to display greater atlantoaxial range of movement and an increased incidence of atlantoaxial or C2/C3 translocation in radiographic examination of the cranium in flexion and extension[33]. Basilar headache, neck pain, and other typical related clinical problems are, however, rare. Atlantoaxial hypermobility has been found to be more common in children with Marfan syndrome than in adults corresponding with the normally occurring stiffening of the ligaments with age[33].

Treatment with bisphosphonates has been shown to improve mobility, level of ambulation, and muscle force in children with moderate to severe OI[34]. The treatment also induces growth in children with OI, as the collapsed vertebral bodies regain a more normal size and shape[35–37]. In the study of Åström and colleagues no child developed scoliosis, kyphosis or basilar impression after the onset of bisphosphonate treatment, and scoliosis remained unchanged in an infant diagnosed with it prior to treatment. In the study, median duration of follow-up was 4.5 years[38]. Anissipour and co-workers found that bisphosphonate treatment started early, before the age of six years, in children with severe OI, decreased significantly the progression rate of the scoliotic curve, whereas in older patients and in patients with milder OI typesbisphosphonates did not seem to have an effect on the progression of scoliosis[11]. The present finding is thus consistent with the previous report on the unchanged overall prevalence of scoliosis following bisphosphonate treatment[11]. In growing patients the occurrence of scoliosis has been found to correlate with low bone mineral density[29]. In contrast, Engelberg and colleagues reported that patients with considerably low bone mineral density in association with moderate and severe OI were diagnosed with both platyspondyly and joint hypermobility but yet did not all develop scoliosis[39]. The group found delayed motor development as a risk indicator for developing scoliosis in untreated children. Hence, the findings on overall effects of bisphosphonate treatment on craniovertebral anomalies are controversial and the protective effect of bisphosphonates on the development of scoliosis is yet to be determined.

The limitations of our study are the relatively small number of patients due to rarity of OI, and the lack of systematic radiographic evaluation of the spine and cranium in extension and flexion. The radiographs had been obtained on clinical indications for diagnostics and planning of treatment to retain the radiation dose of the patients at minimum. Clinical evaluation of scoliosis may leave some mild spinal curvature unnoticed, but it is unlikely that severe scoliosis would have remained undiagnosed. This applies even to joint hypermobility: mild hypermobility may remain undetected while severe forms are recorded. The retrospective nature of our study prevented a more standardized and systematic evaluation of joint hypermobility and scoliosis. The methods used for clinical evaluation of scoliosis and radiological analysis of craniocervical pathology have been previously documented to be reliable[40, 41].

## Conclusions

The standpoint of this paper was to assess how prevalent hypermobility is in patients with OI, and whether hypermobility could, at least partly, explain the less well-known aetiology of the spinal complications in OI. Our present finding that scoliosis and cranial base pathology develop in some patients despite bisphosphonate treatment would indeed suggest that a non-osseous factor, such as ligament laxity, plays an important role. Nevertheless, we found joint hypermobility in 70% of children with OI, and that contrary to spinal complications, it was not over represented in severe forms of OI. Presence of joint hypermobility was not found to correlate with the presence or appearance of either scoliosis or cranial base pathology. Hence, the general severity of the disease remains thus far the best way to predict the development of spinal complications.

## Authors’ original submitted files for images

Below are the links to the authors’ original submitted files for images. Authors’ original file for figure 1
