# Supplementary figures and images for: Association between joint hypermobility, scoliosis, and cranial base anomalies in paediatric Osteogenesis imperfecta patients: a retrospective cross-sectional study
Source: BMC Musculoskelet Disord. 2014 Dec 13;15:428. doi: 10.1186/1471-2474-15-428 (PMC4300610; doi:10.1186/1471-2474-15-428)

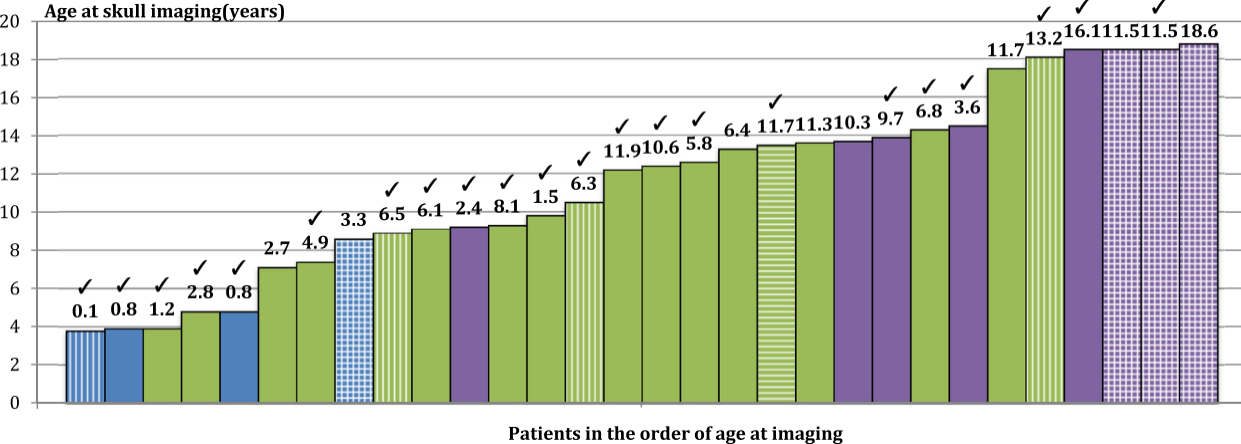

Supplement: Supplementary file 1 — Authors’ original file for figure 1 [file 12891_2014_2387_MOESM1_ESM.pdf]
